# Oxygen desaturation index as alternative parameter in screening patients with severe obstructive sleep apnea

**DOI:** 10.5935/1984-0063.20200119

**Published:** 2022

**Authors:** Lalee Varghese, Grace Rebekah, Priya N, Ashwin Oliver, Regi Kurien

**Affiliations:** 1 Christian Medical College, Vellore, Department of Otorhinolaryngology - Vellore - Tamilnadu - India.; 2 Christian Medical College, Vellore, Department of Biostatistics - Vellore - Tamilnadu - India.; 3 Christian Medical College, Vellore, Department of Pulmonary Medicine - Vellore - Tamilnadu - India.; 4 Christian Medical College, Vellore, Department of Respiratory Medicine - Vellore - Tamilnadu - India.

**Keywords:** Apnea Hypopnea Index, Oxygen Desaturation Index, Obstructive Sleep Apnea, Sleepiness

## Abstract

**Objectives:**

Assess reliability of oxygen desaturation index (ODI) as an alternative parameter to apnea hypopnea index (AHI) in screening patients with severe obstructive sleep apnea (OSA).

**Material and Methods:**

Retrospectively two-year data on demography, anthropometric features, polysomnography (PSG) parameters [AHI, ODI, minimum oxygen saturation (SpO2), mean SpO2], and Epworth sleepiness score (ESS) were collected and analyzed.

**Results:**

Study showed significant correlation of ESS with AHI, ODI, apnea-hypopnea percentage of sleep period time (AH%SPT), mean SpO2 and minimum SpO2 with highest correlation being with AHI. A Cohen’s weighted Kappa analysis showed good concordance of 87.32% between AHI and ODI in classifying severity of OSA, with a significant R^2^ correlation of 0.84 on linear regression. An ODI>20 has a sensitivity of 96.6% and specificity of 69.6% in diagnosing severe OSA.

**Conclusion:**

Good concordance between AHI and ODI makes nocturnal oximetry a less expensive tool to confdently screen patients with severe OSA.

## INTRODUCTION

Sleep-related breathing disorders (SRBD) include a spectrum of conditions ranging from simple snoring, obstructive sleep apnea (OSA), central sleep apnea (CSA), and sleep-related hypoventilation. Obstructive sleep apnea (OSA) is the most common type of SRBD, constituting 90-95% of cases and affecting 6% of men and 4% of women in the general population causing associated excessive daytime sleepiness^1^. OSA is characterized by recurrent episodes of upper airway collapse during sleep leading to complete or partial cessation of airfow, i.e., apnea or hypopnea. If left untreated, the fragmented sleep and intermittent hypoxia in addition to the increased sympathetic nervous activity can lead to wide ranging consequences and impairment of the quality of life in these patients^2^. Excessive daytime sleepiness (EDS) is a leading symptom in these patients where they feel drowsy and sluggish most days, and these symptoms often interfere with work, school, activities, or relationships. Epworth sleepiness score (ESS) is one of the validated methods to assess subjective sleepiness in patients with a clinical suspicion of OSA^3^.

The severity of OSA is quantifed conventionally by the apnea-hypopnea index (AHI), which refects the number of apneas and hypopneas per hour of electroencephalography (EEG) -measured sleep during a full night polysomnography^4^. The most important shortcoming of AHI is that it indicates the number of episodes of events only and does not factor in the duration or depth of abnormal respiratory events, i.e., the severity of oxygen desaturation associated with apnea or hypopnea. When the depth and duration of apnea or hypopnea increase, the number of respiratory events happening in an hour may paradoxically fall contrary to the increased severity of the episodes.

Since the intermittent desaturations play a main role in the development of OSA related complications, yet another parameter that can be considered to grade the severity of OSA is oxygen desaturation index (ODI)^5^. ODI refers to the average number of desaturation episodes occurring per hour, where desaturation episodes are defined as a decrease in the mean oxygen saturation of ≥3% (over the last 120 seconds) that lasts for at least 10 seconds.

The aim of the present study was to determine the correlation and concordance between AHI and ODI in assessing severity of OSA and to evaluate reliability of oxygen desaturation index (ODI) as an alternative parameter to apnea hypopnea index (AHI) in screening patients with severe obstructive sleep apnea (OSA). Factors affecting patients’ sleepiness as measured by ESS were also secondarily analyzed.

## MATERIAL AND METHODS

This is a retrospective study including all consecutive patients diagnosed to have OSA based on polysomnography (PSG) over a period of two years. Only those patients with an AHI of >5 events/h, with >50% apneic events being obstructive were considered to have obstructive apnea. Patients with predominant central apnoea and those less than 18 years were excluded from the study. Institutional review board clearance (IRB No.: 12096) was obtained for the study. Data collected included demography (age, gender, and comorbidities), anthropometric data (body mass index or BMI, neck circumference), and clinical data on subjective sleepiness based on Epworth sleepiness score (ESS) and sleep study parameters which included AHI, ODI, apnea-hypopnea percentage of sleep period time (AH%SPT), minimum SpO_2_ and mean SpO_2_. Full night polysomnography recorded signals including electrocardiogram (ECG), electromyogram (EMG), electrooculogram (EOG), electroencephalogram (EEG) and from abdomen, chest leads, and nasal fow. A Masimo LNCS DCI adult reusable clip pulse oximeter was used to record oxygen saturation with a maximum sampling rate of 2,000Hz and storage rate of 3Hz.

Patients were categorized into mild, moderate and severe OSA based on the AHI values of 5-15, 15-30, and ≥30 events per hour, respectively. Desaturation episodes were defined as a decrease in the mean oxygen saturation of ≥3% for more than 10 seconds and ODI was calculated based on the number of desaturation episodes per hour. ODI was graded into three groups: mild (5-15), moderate (15-30), and severe (≥30) OSA. Patients with an ODI<5 were graded as having no oxygen desaturation.

Epworth sleepiness score (ESS) of >10 was considered as clinically significant sleepiness.

### Statistical analysis

The demographic, anthropometric and summary of data from the PSG were presented with descriptive statistics. Categorical data were presented as frequency with percentage. The mean±SD was used for continuous data with normal distribution. Correlation between AHI and ODI and the various factors affecting ESS were analyzed using Pearson’s correlation coefficient. The severity of OSA classifed using ODI and AHI was cross-tabulated, and differences in distribution were examined using the χ^2^-test. Scatter plot, Bland-Altman plot and Cohen’s Weighted Kappa (κ) coefficient were used to check the concordance between AHI and ODI in classifying OSA severity. Receiver operating characteristic curve analysis was done to arrive at the threshold of ODI with respect to the severity of AHI.

## RESULTS

A total of 142 subjects were included in this study. The mean age of the cohort was 42 years (range 23-68) with male to female ratio of 9:1. Hypertension was the main comorbidity seen in the group (41 patients, 28.1%), the others being dyslipidemia (11.6%), diabetes mellitus (10.3%), and hypothyroidism (7.5%).

The BMI of the cohort ranged from 18.9 to 45 with a mean of 28.2. 25 patients (17.6%) were of normal weight whereas 76 (53.5%) and 41 patients (28.9%) were overweight and obese, respectively. The neck circumference (NC) of the cohort ranged from 31 to 49 with a mean of 40.13cms.

The study group showed ESS ranging from 0 to 24 with a mean of 10.49. The mean value of minimum SpO_2_ was 74.7. Average of the mean SpO_2_ value during PSG was 88.19.

AHI varied from 6 to 98 with mean of 42.32. Based on AHI, 87 (61.26%) patients had severe OSA whereas the severity of OSA was mild in 19 patients (13.38%) and moderate in 36 (25.35%) patients. The mean ODI of the study group was 40.43 ranging from 4.9 to 96.8. When classifed based on ODI, 29 (20.42%) patients came under mild OSA whereas 36 (25.35%) and 77 (54.22%) patients had moderate and severe OSA, respectively. The mean difference between AHI and ODI was 1.344, 3.083, and .539 in the normal weight, overweight, and obese category, respectively.

Of the patients classifed as mild OSA based on the AHI grading, majority (89.5%) were graded as mild and 10.5% patients as moderate OSA based on ODI. 50% patients with moderate OSA were graded as moderate using both parameters. However, among the remaining 50%, 33.3% and 16.7%, respectively, were graded as mild and severe according to ODI. In the severe OSA group, 81.6% fell in the severe grade based on ODI also (Table 1).

**Table 1:** AHI and ODI correlation according to the OSA grades.

					AHI * ODI				
			ODI		Total	Correlation coefficient	Agreement	Kappa	*p*-value
		Mild OSA	Moderate OSA	Severe OSA					
**AHI**	**Mild OSA**	17 (89.5%)	2 (10.5%)	0 (0.0%)	19				
	**Moderate OSA**	12 (33.3%)	18 (50.0%)	6 (16.7%)	36	.919	87.32%	0.672	<0.001
	**Severe OSA**	0 (0.0%)	16 (18.4%)	71 (81.6%)	87				
**Total**		29	36	77	142				

**Figure 1:**
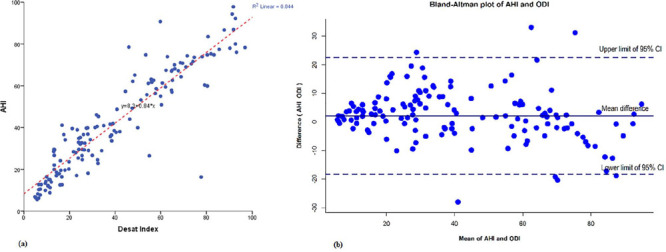
A. Scatter plot showing apnea hypopnea index (AHI) versus oxygen desaturation index (ODI) with linear regression and 95% confdence interval. R2 linear 0.844, AHI=0.84 x ODI + 8.3; B. Bland-Altman plot of apnea hypopnea index (AHI) and oxygen desaturation index (ODI) where, X axis represents the average of AHI and ODI and the Y axis represents the difference of AHI and ODI (AHI-ODI). When the average of AHI and ODI is very low or high, the ODI tended to overestimate AHI.

In a Cohen’s weighted Kappa analysis, the Kappa and the proportional agreement observed between AHI and ODI in classifying severity of OSA was 0.67 and 87.32%, respectively (Table 1). The relationship between the ODI and AHI is further depicted in Figure 1.

Receiver operating characteristic curve analysis was done to arrive at the threshold of ODI with respect to the severity of AHI. The predictive performance of different ODI cut-off points for severe OSA is shown in Table 2. ODI>15 had 100% sensitivity, 53.6% specificity, positive predictive value (PPV) of 77%, and 100% negative predictive value (NPV). Sensitivity to detect patients with severe OSA was 96.6% at ODI 20 and 89.7% at ODI 25.

**Table 2:** Predictive value of different ODI cutoffs for severe OSA.

AHI>30/hr	Sensitivity (%)	Specificity (%)	PPV (%)	NPV (%)
ODI>15	100	53.6	77	100
ODI>20	96.6	69.6	83.2	92.9
ODI>25	89.7	78.6	86.7	83.0

Notes: NPV = Negative predictive value; PPV = Positive predictive value.

The correlation between various factors contributing to sleepiness was looked into. A statistically significant correlation of ESS was seen with AHI, ODI, AH%SPT, mean SpO_2_ and minimum SpO_2_. This was consistent even when analyzed as groups, ESS<10 and >10 also (Table 3). However the strength of association was strongest with AHI (r=.409) than with AH%SPT and ODI (r=0.379 and 0.340, respectively). Mean SpO_2_ showed a stronger negative correlation to ESS (r=-.419) than minimum SpO_2_ (r=-.376). Sex, BMI, and NC failed to show any significant correlation with ESS scores. None of the co morbidities showed any correlation to ESS.

**Table 3:** Correlation of different parameters to ESS.

Variable	ESS Mean±SD n (%)		p-value
	**≤10**	**>10**	
**Sex**			
Male	63(48.8)	66(51.2)	0.303
Female	10(62.5)	6(37.5)	
**BMI**			
<25	15(55.6)	12(44.4)	0.363
25-30	34(44.7)	42(55.3)	
>30	24(57.1)	18(42.9)	
**NC**	39.69±3.37	40.57	0.095
**AHI**			
Mild	12(60.0)	8(40.0)	0.008
Moderate	25(69.4)	11(30.6)	
Severe	35(40.2)	52(59.8)	
**ODI**			
Mild	20(69.0)	9(31.0)	0.006
Moderate	22(61.1)	14(38.9)	
Severe	29(38.2)	47(61.8)	
**AH%SPT**	22.22±14.93	33.03±19.80	<0.01
**Mean SpO2**	89.94±2.99	86.42±6.09	<0.01
**Minimum SpO2**	78.38±9.12	71.08±13.22	<0.01

## DISCUSSION

Apnea-hypopnea index is currently considered as the investigation of choice for the estimation of the severity of OSA. Cohen’s weighted Kappa analysis in our study showed a good level of concordance (k=0.67) and proportional agreement (87.32%) between AHI and ODI (*p*<0.001) in predicting the severity of OSA. The Bland-Altman plot depicts that at very high and low AHI values the points are below zero showing ODI overestimates AHI. In the rest of the plot, the points are equally scattered above and below zero suggesting that there is no consistent difference between the two measurements of severity of OSA. This is more emphasized by the R^2^ value of 0.84 in the linear regression plot. These results are in agreement with that of a study by Temirbekov et al. (2018)^5^ where a concordance of 72.3% between AHI and ODI was observed. In view of the above findings, we postulate that ODI could be used as a useful alternative parameter for determining the severity of OSA.

In a study by Chung et al. (2012)^6^, ODI >5, ODI >15, and ODI>30 were found to be good predictors for AHI>5, AHI>15, and AHI>30, respectively. Similarly, Hang et al. (2015)^7^ concluded that overnight pulse oximetry is diagnostic in detecting severe OSA. In our study since the sensitivity of ODI to detect patients with severe OSA was 100%, 96.6%, and 89.7% at levels ODI 15, 20, and 25, respectively, we can confdently exclude the possibility of severe OSA if a patient had ODI<15. If one cutoff of ODI needs to be chosen, ODI>20 would be a good choice as it could detect almost all patients with severe OSA, while still retaining a reasonable specificity.

Patients with SRBD require regular assessments of the severity of OSA especially when they are on conservative measures like weight reduction and oropharyngeal exercises. This holds true even for patients who have undergone surgical interventions who also require serial follow up. Although PSG is the gold standard, financial implications and limited availability of time slots for a routine full night polysomnography restricts its frequent use^6^. Another option recommended by AASM is use of type III portable home device, the availability of which is limited in developing countries. In this scenario nocturnal oximetry using ODI as shown in this study, could be used as an effective alternative parameter to grade severity of OSA. Only patients with moderate and severe OSA based on ODI can be planned for a routine PSG.

Excessive daytime sleepiness (EDS) is a well acknowledged consequence of obstructive sleep apnea (OSA)^8^. Multiple sleep latency test and maintenance of wakefulness test are objective measures of sleepiness but are complex and costly to use. ESS was adopted for assessment of EDS in our study since it has a statistically significant association though weak correlation with mean sleep latency (r=0.51, *p*>0.01)^9^.

Analysis of data demonstrated that the differences in ESS are unrelated to the anthropometric characteristics like neck circumference (*p*-value .095) and comorbid conditions of the patients. Despite 82.4% subjects being overweight or obese, the correlation of BMI with sleepiness in our study was also not significant (*p*-value .363).

AHI used for assessing severity of OSA does not consider the morphology or duration of the breathing cessations and subsequent oxygen desaturations. Longer apneas and deeper desaturations may have more severe consequences like EDS than shorter and shallower ones. To address these issues, we looked at the relation of ESS to AHI and other respiratory parameters.

A statistically significant correlation was observed between the subjective self-assessment of daytime sleepiness on the standardized ESS questionnaire and the objective respiratory parameters AHI, ODI, AH%SPT, mean SpO_2_ and minimum SpO_2_, measured during PSG. There are studies in literature which have shown evidence in favour of such a positive correlation between AHI and ESS^1,10^ whereas some provided evidence against the same^11,12,13^.

In this study, mean SpO_2_ and minimum SpO_2_ showed a mild negative correlation with ESS, which was statistically significant. In a study by Bausmer et al. (2010)^11^ there was no correlation between minimum SpO_2_ and ESS.

Yet another area where this less expensive alternative investigation may be useful is in preoperative screening of patients with history of snoring scheduled for any non-OSA related elective surgery. In case the desaturation index is high, adequate precautions can be taken by the anaesthetist and operating team in the perioperative period. This may be applicable in smaller centres where facilities and expertise for a full night PSG may not be easily available.

The limitations of our study are its retrospective nature, low proportion of women, fewer subjects with mild OSA, and non-inclusion of patients with AHI less than 5. However, our study highlights the utility of ODI as an easy to use, less expensive tool to confdently screen patients who will not require an elaborate PSG. Only those patients with ODI of greater than 20 need to be subjected for further evaluation. This will help optimize the sleep study appointments and reduce the financial burden on patients.

## CONCLUSION

Good concordance between AHI and ODI makes nocturnal oximetry a less expensive tool to confdently screen patients with severe OSA. Those patients with ODI greater than 20 need to be subjected for further PSG evaluation. There is a significant correlation between the subjective self-assessment of daytime sleepiness on the standardized ESS questionnaire and the objective respiratory parameters AHI, ODI, AH%SPT, mean SpO2 and minimum SpO2, measured during PSG.
